# Research progress on the roles of actin-depolymerizing factor in plant stress responses

**DOI:** 10.3389/fpls.2023.1278311

**Published:** 2023-11-16

**Authors:** Yongwang Sun, Mengmeng Shi, Deying Wang, Yujie Gong, Qi Sha, Peng Lv, Jing Yang, Pengfei Chu, Shangjing Guo

**Affiliations:** School of Agricultural Science and Engineering, Liaocheng University, Liaocheng, China

**Keywords:** actin-depolymerizing factor, microfilament, plant growth and development, biotic stress, abiotic stress, stress response, research progress

## Abstract

Actin-depolymerizing factors (ADFs) are highly conserved small-molecule actin-binding proteins found throughout eukaryotic cells. In land plants, ADFs form a small gene family that displays functional redundancy despite variations among its individual members. ADF can bind to actin monomers or polymerized microfilaments and regulate dynamic changes in the cytoskeletal framework through specialized biochemical activities, such as severing, depolymerizing, and bundling. The involvement of ADFs in modulating the microfilaments’ dynamic changes has significant implications for various physiological processes, including plant growth, development, and stress response. The current body of research has greatly advanced our comprehension of the involvement of ADFs in the regulation of plant responses to both biotic and abiotic stresses, particularly with respect to the molecular regulatory mechanisms that govern ADF activity during the transmission of stress signals. Stress has the capacity to directly modify the transcription levels of *ADF* genes, as well as indirectly regulate their expression through transcription factors such as MYB, C-repeat binding factors, ABF, and 14-3-3 proteins. Furthermore, apart from their role in regulating actin dynamics, ADFs possess the ability to modulate the stress response by influencing downstream genes associated with pathogen resistance and abiotic stress response. This paper provides a comprehensive overview of the current advancements in plant *ADF* gene research and suggests that the identification of plant *ADF* family genes across a broader spectrum, thorough analysis of *ADF* gene regulation in stress resistance of plants, and manipulation of *ADF* genes through genome-editing techniques to enhance plant stress resistance are crucial avenues for future investigation in this field.

## Introduction

Plants are sessile growth organisms that face various unfavorable biotic and abiotic stresses during their life cycle (Verma et al., 2016). Biotic and abiotic stresses refer to biological or environmental factors that are detrimental to the survival and growth of plants at all phenological stages and can cause significant damage to agricultural production (Zhu, 2016; Li et al., 2019). Plants use numerous physiological and biochemical mechanisms to mitigate the impact of adverse conditions on their growth and survival (Hasanuzzaman et al., 2013; Zhao et al., 2020).

The actin cytoskeleton is an essential component of the plant cellular skeletal system. It not only maintains the shape of the cell by providing it with a three-dimensional structure but also participates in regulating various life activities, including cell motility, growth, division, differentiation, organelle movement, endocytosis, exocytosis, and responses to physiological and environmental signals (Staiger, 2000; Pollard and Cooper, 2009; Henty-Ridilla et al., 2013; Wang and Mao, 2019). In cells, actin exists in two forms, i.e., globular actin (G-actin), which is generally present as a monomer, and filamentous actin (F-actin), which exists as polymerized filaments (also known as microfilaments). The latter is the form that primarily performs biological functions (Staiger, 2000; Pollard, 2016). During microfilament formation, three G-actin molecules initially aggregate to form a nuclei (called nucleation), following which other G-actin molecules are gradually added to the ends of the nuclei to elongate the filament (called elongation) (Pollard and Cooper, 2009). The elongation rates considerably differ between the two ends, with the faster-elongating end termed the “barbed end” and the slower-elongating end termed the “pointed end” (Li et al., 2015). Different microfilaments subsequently crosslink to form a three-dimensional network structure or align in parallel to each other to form thicker bundles of microfilaments (Uribe and Jay, 2009).

In response to physiological or environmental signals, the two cellular forms of actin constantly polymerize and depolymerize, resulting in highly dynamic changes in microfilaments, ensuring a rapid cellular response (Pollard and Cooper, 2009). The microfilaments and their dynamic changes play an important role in regulates plant stress tolerance (Li et al., 2015; Porter and Day, 2016; Wang et al., 2022). For example, when actin polymerization is blocked with the inhibitor latrunculin B, plants are more susceptible to pathogenic and nonpathogenic bacteria (Henty-Ridilla et al., 2013), and numerous studies have also revealed that actin dynamics correlated with the plant response to abiotic stress, such as cold (Pokorna et al., 2004), heat (Müller et al., 2007; Malerba et al., 2010), salt (Wang et al., 2010), and alkaline (Zhou et al., 2010). Understanding the regulation of microfilament dynamics will enrich our understanding of plant stress response.

Actin polymerization, depolymerization, crosslinking, and bundling are processes regulated by a series of actin-binding proteins (ABPs), and hundreds of ABPs have been discovered in eukaryotes (Li et al., 2015; Porter and Day, 2016). In general, ABPs interact with actin and regulate their dynamic changes, thereby participating in various physiological activities of the cell (Pollard, 2016; Augustine et al., 2021). Actin-depolymerizing factor (ADF) is a small-sized (15-22 kDa) and highly conserved ABP ubiquitously exist in eukaryotic cells (Maciver and Hussey, 2002). The first ADF was isolated from chicken embryo brain cells, and the authors found that the isolated protein is distinct from other ABPs in its isoelectric point and has the capacity to depolymerize F-actin (Bamburg et al., 1980). Subsequently, *ADF* genes have been cloned from various eukaryotes, including fungi, animals and plants (Maciver and Hussey, 2002; Inada, 2017). ADF can bind both G-actin and F-actin and regulate remodeling of microfilament framework via its specialized biochemical activities (Hussey et al., 2002; Andrianantoandro and Pollard, 2006). Initially, ADF was found to severs or depolymerizes F-actin into shorter fragments or G-actin monomers, which provides new sites for actin filament initiation and supplies additional actin monomers for further polymerization (Bamburg et al., 1980; Maciver et al., 1991; Carlier et al., 1997). Subsequently, the biochemical activity of ADF was found to depend on the local concentration in cells (Andrianantoandro and Pollard, 2006). Low concentrations of ADF favor severing or deploymerizing whereas high concentrations favor actin nucleation as well as accelerate P_i_ release from ADP-P_i_ subunits in filaments and dissociation of branches formed by actin-related protein2/3 complex (Blanchoin and Pollard, 1999; Blanchoin et al., 2000; Andrianantoandro and Pollard, 2006). This range of biochemical activities makes ADF an important factor for regulating dynamic changes in actin filaments, which involves in most of the cellular processes of eukaryotes (Staiger, 2000; Pollard, 2016). Hence, ADFs widely participates in numerous plant growth and development processes, including flowering (Burgos-Rivera et al., 2008), pollen development and pollen tube growth (Chen et al., 2002; Daher and Geitmann, 2012; Zheng et al., 2013), cell elongation and secondary cell wall formation (Wang et al., 2009a), and responses to various biotic and abiotic stresses (Huang et al., 2012; Tang et al., 2016; Inada, 2017; Zhang et al., 2017).

Previous studies have reviewed plant *ADF* family genes in terms of evolutionary classification, expression profiles, transcriptional regulation, biochemical activity, and biological function (Hussey et al., 2002; Maciver and Hussey, 2002; Inada, 2017). However, there are many papers published and significant progress has been made since then, especially those regarding the molecular mechanisms underlying its involvement in signal responses to stress conditions. Recently, the publication of new plant genome sequences has led to the systematic reporting of *ADF* gene families from a dozen plant species (Feng et al., 2006; Ruzicka et al., 2007; Huang et al., 2012; Khatun et al., 2016; Huang et al., 2020; Xu et al., 2021; Sun et al., 2023). Furthermore, research on plant *ADF* genes and their involvement in stress responses has gradually received more attention, with significant progress being made in recent years. In this paper, we reviewed the research progress of the responses and molecular regulatory mechanisms of plant *ADF* genes to different forms of biotic and abiotic stresses. This review aims to provide a thorough understanding of the role played by *ADF* genes in plant stress responses and the molecular regulatory mechanisms that underlie them, and offers suggestions for future research directions in this field.

## Expression profiles and biochemical activity diversification of plant *ADF* genes

### Varying numbers of *ADF* genes in different species

Although *ADF* genes exist in all eukaryotes, the number of these genes considerably varies among species. Single-cell eukaryotes and animal genomes contain no more than three members of the ADF family. For example, yeast (*Saccharomyces cerevisiae*), roundworm (*Caenorhabditis elegans*), and the alga *Chlamydomonas reinhardtii* all have only one *ADF* gene (Gunning et al., 2015). Three *ADF* members are present in the genomes of zebrafish (*Danio rerio*), jungle fowl (*Gallus gallus*), and humans (*Homo sapiens*) (Gunning et al., 2015). Conversely, land plants possess an expanded *ADF* gene family. For example, eleven *ADF* genes have been identified in *Arabidopsis thaliana* (Feng et al., 2006; Ruzicka et al., 2007), rice (*Oryza sativa*; Feng et al., 2006; Huang et al., 2012), and tomato (*Solanum lycopersicum*; Khatun et al., 2016) each; eight in cucumber (*Cucumis sativus*; Liu et al., 2016) and Antarctic hairgrass (*Deschampsia antarctica*; Byun et al., 2021) each; nine in common bean (*Phaseolus vulgaris*; Ortega-Ortega et al., 2020); ten in pigeon pea (*Cajanus albicans*; Cao et al., 2020); thirteen in maize (*Zea mays*; Huang et al., 2020); fourteen in poplar (*Populus trichocarpa*; Roy-Zokan et al., 2015); eighteen in soybean (*Glycine max*; Sun et al., 2023); twenty-five in wheat (*Triticum aestivum*; Xu et al., 2021); twenty-seven in banana (*Musa acuminata*; Nan et al., 2017); and thirty-seven in upland cotton (*Gossypium hirsutum*; Sun et al., 2021). In contrast to single-cell eukaryotes and animals, plants exhibit a multitude of distinct and functionally specialized actin filament systems, alongside a larger actin gene family (McDowell et al., 1996; Zhang et al., 2010; Gunning et al., 2015). Likewise, an increased number of genes has been observed in numerous ABP gene families, such as profilin, formin, and villin (Bao et al., 2011; Gunning et al., 2015; Duan et al., 2021; Zhou et al., 2023). The diverse members within these extensive gene families, believed to have originated from gene duplication events, are presumed to be expressed in a highly differential manner, specific to tissues, environmental conditions, and temporal factors (McDowell et al., 1996; Bao et al., 2011). This expression pattern enables plants to dynamically restructure the actin cytoskeleton in response to evolving requirements throughout their growth and development processes (Gunning et al., 2015). Regarding plant *ADF* genes, the expansion of the gene family may facilitate their expression in intricate biological profiles, enabling differentiation into various biological functions, as elucidated in numerous subsequent articles.

### Expression profiles of *ADF* genes in *Arabidopsis*

Among all land plants, the expression characteristics and biological functions of *ADF* genes in *Arabidopsis* have been the most extensively studied. Phylogenetic analysis reveals that the eleven *AtADF* genes can be divided into four groups (I–IV), with group II further divided into subgroups II-a and II-b (Feng et al., 2006; Ruzicka et al., 2007). Within each group, *AtADF* genes demonstrate comparable tissue-specific expression patterns, although notable disparities in expression characteristics exist among members across distinct groups. Group I comprises four genes: *AtADF1*, *AtADF2*, *AtADF3*, and *AtADF4*. These genes are stably expressed at high levels in all plant tissues/organs except in pollen. Overall, *AtADF3* exhibits the highest expression level. Group II comprises four genes: *AtADF7*, *AtADF8*, *AtADF10*, and *AtADF11*, which are preferentially expressed in cell types demonstrating polarized growth characteristics. *AtADF7* and *AtADF10* are members of the subgroup II-a and are specifically expressed in mature pollen grains and pollen tubes, whereas *AtADF8* and *AtADF11* (subgroup II-b) are specifically expressed in root hairs and root epidermal cells that can differentiate into root hairs. Group III comprises only two genes, *AtADF5* and *AtADF9*, which exhibit lower expression levels in vegetative tissues but are highly expressed in cells undergoing rapid growth or differentiation, including callus tissues, young leaves, and meristematic regions. Group IV only contains one gene, *AtADF6*, which is stably expressed in all tissues, including pollen (Ruzicka et al., 2007). In a study by Dong et al. (2001), *ADF* gene promoter–GUS fusions were employed for genetic transformation in *Arabidopsis*, and the authors found that *AtADF1* and *AtADF6* were expressed in the vascular tissues of all organs, while *AtADF5* was only expressed in the root apical meristem. Immunocytochemical analysis further revealed that proteins encoded by group I genes are localized to the nucleus and cytoplasm simultaneously, while proteins encoded by group II genes are mainly localized to the cytoplasm of pollen tubes and the apical regions of root hairs (Ruzicka et al., 2007). These results indicate that the expression and localization of ADFs are precisely regulated, and different ADFs are required to function in distinct tissue types and subcellular locations.

### Expression profiles of *ADF* genes in several crops

Previous studies have reported that *ADF* genes in other plants exhibit tissue-specific expression characteristics similar to those found in *Arabidopsis*. Here we take the expression patterns of *ADF* genes in several crops, including rice, maize, wheat, cotton, tomato, and soybean, as examples. *OsADF2*, *OsADF4*, *OsADF5*, and *OsADF11* are persistently expressed in the roots, stems, leaves, sheaths, spikelets, and seeds of rice, while *OsADF9* is specifically expressed in spikelets during the heading stage (Huang et al., 2012). *ZmADF3*, *ZmADF4*, *ZmADF5*, *ZmADF6* and *ZmADF10* showed relatively higher expression in all tissues of maize, whereas *ZmADF1*, *ZmADF2*, *ZmADF7*, *ZmADF12*, and *ZmADF13* showed high expression levels in reproductive organs such as tassel, anther, and pollen (Huang et al., 2020). Of the twenty-five *TaADF* genes in wheat, nine of them exhibit anther-specific expression, while the others are diversely expressed in different tissues (Xu et al., 2021). In upland cotton, *GhADF6* and *GhADF8* are predominantly expressed in petals while *GhADF7* is highly expressed in anthers (Li et al., 2010). Among the nine *SlADF* genes in tomato, *SlADF1*, *SlADF3* and *SlADF10* are predominately expressed in flowers and specifically in the stamen compared to other parts (Khatun et al., 2016). In soybean, our lab used genome-wide identification techniques to show that the soybean *ADF* gene family displays tissue-specific expression patterns very similar to those found in *Arabidopsis* (Sun et al., 2023). In short, *GmADF* genes in groups I and IV are expressed throughout the soybean plant, those of group II are specifically expressed in flowers, while the expression level of genes in group III is lower than that in groups I and IV (Sun et al., 2023).

### Biochemical activity diversification of plant *ADF*s

The expansion and diversification of the expression patterns of *ADF* gene family members in land plants imply that their biochemical activities or biological functions may have been differentiated during evolution (Ren and Xiang, 2007; Tholl et al., 2011). Biochemical experiments have shown that nine AtADF members in groups I, II, and IV of *Arabidopsis* can sever or depolymerize F-actin, with the four members of group I being the most active (Nan et al., 2017). The two AtADF members in group III do not show severing or depolymerizing activities, but instead have the ability to promote F-actin bundling (Tholl et al., 2011; Nan et al., 2017). Three crucial amino acid alterations were confirmed to be responsible for these divergent biochemical activities. Taking AtADF9 from the group III as an example, the 3^rd^ Leu, 4^th^ Lys, and 18^th^ Lys (the corresponding amino acid residue in AtADFs from other three groups are Met, Ala, Leu/Thr, respectively) are necessary for its F-actin bundling activity (Nan et al., 2017). By comparing variations in the amino acid sequences of the *Arabidopsis* protein and its homologs in other plants, Nan et al. (2017) suggested that this biochemical activity divergence may be widely present in angiosperms.

## Function of plant *ADF* genes in biotic stress

Biological stress of plants refers to the inhibition of their growth, development, and survival caused by biological factors such as pests, bacteria, fungi, viruses, etc. (Verma et al., 2016; Jiang et al., 2017). These harmful animals or microbes attack numerous agricultural crops, causing devastating effects on plant productivity and yield (Leonard et al., 2017). Increasing studies showed that plant *ADF* genes and actin cytoskeleton dynamics are widely involved in plant responses to biotic stress (**Table 1**). Understanding the biological function and the regulatory mechanism of these ADFs is essential for the development of biotic stress-tolerant crops. In this section, we will summarize the research progress of *ADF* gene in plant response to biotic stress.

**Table 1:** *ADF* genes involved in biotic stress whose functions have been elucidated.

Gene	Organism	Inducing factor	Function	Upstream regulator or downstream target	Reference
*AtADF2*	*Arabidopsis*	Root-knot nematode	Downregulation of *AtADF2* enhances plant tolerance of nematodes	Not given (NG)	Clément et al., 2009
*AtADF3*	*Arabidopsis*	Green peach aphid	Required for limiting green peach aphid infestation	Positively regulate *PAD4* expression	Mondal et al., 2018
*AtADF4*	*Arabidopsis*	Powdery mildew	Negative regulator of plant resistance to powdery mildew	NG	Inada et al., 2016
*AtADF6*	*Arabidopsis*	Powdery mildew	Negative regulator of plant resistance to powdery mildew	Inhibit the function of RPW8.2	Wang et al., 2009b
*HvADF3*	Barley	Powdery mildew	Negative regulator of plant resistance to powdery mildew	NG	Miklis et al., 2007
*AtADF4*	*Arabidopsis*	*Pseudomonas syringae* (*Pst*)	Positive regulator of *Pst* resistance	Positively regulate *RPS5* expression	Tian et al., 2009
*GhADF6*	Cotton	Verticillium wilt	Negative regulator of *Verticillium* wilt resistance	NG	Sun et al., 2021
*PvADFE*	Common bean	Rhizobia	Negative regulator of *Rhizobium* inoculation	NG	Ortega-Ortega et al., 2020
*GmADF2*	Soybean	Soybean mosaic virus	Negative regulator of SMV resistance	Interacts with SMV-P3	Lu et al., 2015
*TaADF3*	Wheat	Stripe rust	Negative regulator of Stripe rust resistance	NG	Tang et al., 2016
*TaADF4*	Wheat	Stripe rust	Positive regulator of Stripe rust resistance	NG	Zhang et al., 2017
*TaADF7*	Wheat	Stripe rust	Positive regulator of Stripe rust resistance	NG	Fu et al., 2014

### Pest resistance

#### Resistance to root-knot nematode

The root-knot nematode (*Meloidogyne incognita*) is a highly specialized and polyphagous plant-pathogenic nematode. Its second-stage juveniles can penetrate plant root apical meristems via stylets. Thereafter, they migrate within the plant and establish parasitic relationships with vascular tissues, leading to the formation of giant cells and production of galls (Fuller et al., 2008). Moreover, the cytoskeletal system of giant cells undergoes rearrangement during its altered development (Jammes et al., 2005). The expression levels of five *ADF* genes (i.e., *AtADF2*–*AtADF6*) were higher in the galls of infected roots compared with those of an uninfected control. Of these genes, *AtADF2* exhibited a nearly three-fold increase in expression 2–3 weeks following nematode infection, and its expression was concentrated in giant cells. Moreover, *AtADF2* knockdown in *Arabidopsis* increased the bundling of actin filaments, resulting in delayed giant cell development and decreased nematode reproduction. Thus, these findings imply that *AtADF2* positively regulates plant resistance to root-knot nematodes (Clément et al., 2009). Similarly, in cucumber five of eight *CcADF* genes demonstrated increased expression in nematode-induced galls, suggesting that *CcADF* genes may facilitate nematode feeding on cucumber roots (Liu et al., 2016).

#### Resistance to aphids

Aphids (Hemiptera: Aphididae), a diverse family of ~250 different species, are pests who feed on plants and affect plant growth and productivity via removing nutrients from sieve elements, altering source–sink relationships, and spreading viral diseases (Goggin, 2007). *Arabidopsis atadf3* mutants were more susceptible to green peach aphids (GPAs; *Myzus persicae* Sülzer) infestation compared with wild type plants. GPAs fed faster and for a longer duration on *atadf3* mutants, and their populations could therefore reproduce more quickly. Introducing *AtADF3* into *atadf3* mutant plants rescued the resistance to GPAs, indicating that *AtADF3* has a critical role in limiting GPAs infestation (Mondal et al., 2018). By monitoring aphid feeding behavior, the authors found that the *AtADF3* expression hinders with the ability of GPAs to find and feed from sieve elements. PAD4 (phytoalexin-deficient 4) is an important regulatory factor in *Arabidopsis* defense against peach aphids, negatively regulating aphid feeding and fecundity (Louis et al., 2010). Further research confirmed that PAD4 is a critical downstream player of the *AtADF3*-dependent defense mechanism (Mondal et al., 2018).

#### Resistance to corn borer

The corn borer is a major maize pest in many regions of the world, where it severely affects its yield by feeding on organs such as leaves, stems, and male and female inflorescences (Meihls et al., 2012). A recent genome-wide association analysis revealed that *ZmADF4* is significantly associated with resistance to the Mediterranean corn borer (*Sesamia nonagrioides*) in the stem (Samayoa et al., 2015). The biological function of *ZmADF4* in maize resistance to corn borer is worth further exploration.

### Fungal stress

#### Resistance to powdery mildew

Powdery mildew is a obligate biotrophic fungal pathogen that seriously threatening over 10,000 plant species, including crops, vegetables, trees, and ornamental plants (Hirose et al., 2005; Hückelhoven and Panstruga, 2011). *ADF* genes from different plants have been found to play different roles in regulating powdery mildew resistance. In *Arabidopsis*, the four Group I *AtADF* genes have been found to play a negative regulatory role regarding resistance to *Golovinomyces orontii* (*G.orontii*), with *AtADF4* exhibiting the most significant effect. In *atadf4* mutants and *atadf1-4* quadruple knockdown plants, researchers identified an accumulation of hydrogen peroxide and cell-specific death at the sites of *G.orontii* infection. In addition, they also found an increase in the abundance of microfilaments, and the plants show enhanced resistance against powdery mildew (Inada et al., 2016). RPW8.2 (resistance to powdery mildew 8.2) is an atypical mildew resistance protein found in *Arabidopsis* (Xiao et al., 2001). Overexpression of *AtADF6* (belongs to group IV) inhibited its localization to the membrane surrounding the powdery mildew fungal haustorium, which is required for inducing resistance against powdery mildew. Thus, this evidence indicates that *AtADF6* may play a negative role toward powdery mildew resistance (Wang et al., 2009b). In contrast, the overexpression of *AtADF5* (belongs to group III) had no effect on RPW8.2 localization, implying the existence of functional diversification among ADF members in plant response to powdery mildew (Wang et al., 2009b). It is reasonable to presume that the functional diversification between *AtADF5* and *AtADF6* may resulted from their difference in biochemical activity (Nan et al., 2017) or expression profile (Dong et al., 2001). Ectopic expression of *HvADF3* in barley (*Hordeum vulgare*) epidermal cells was found to disrupt the integrity of the actin cytoskeleton in cells. This in turn enhanced fungal entry and lead to increased susceptibility to the barley pathogen *Blumeria graminis* f. sp. *hordei* (*Bgh*) (Miklis et al., 2007). Moreover, transient overexpression of *AtADF1*, *AtADF5*, *AtADF6*, *AtADF7*, and *AtADF12* was found to significantly increase the entry rate of *Bgh* in barley, while the overexpression of *AtADF2*, *AtADF3*, *AtADF4* and *AtADF9* had no significant effect.

#### Resistance to stripe rust

In wheat, stripe rust caused by *Puccinia striiformis* f. sp. *tritici* is a widespread and devastating disease (Hovmøller, 2007). Different wheat *ADF* genes were found to exhibit varied response patterns against different physiological races of this pathogen, and may therefore play different roles in regulating stripe rust resistance. For example, the avirulent race CYR23 strongly induced the expression of *TaADF4* and *TaADF7* in wheat, while the virulent race CYR31 induced their expression to a lesser extent. Silencing *TaADF4* and *TaADF7* in wheat lines inoculated with CYR23 led to significant changes in microfilament structures, reduced accumulation of reactive oxygen species (ROS), and weakened hypersensitive reactions. Taken together, these effects indicate that *TaADF4* and *TaADF7* positively regulate wheat resistance against non-adapted races of stripe rust by modulating microfilament dynamics (Fu et al., 2014; Zhang et al., 2017). In contrast to the response patterns of these genes, *TaADF3* showed elevated expression levels upon CYR31 induction but showed significantly decreased expression levels following CYR23 inoculation. Silencing *TaADF3* enhanced wheat resistance to CYR31 while reducing both ROS accumulation and hypersensitive reactions (Tang et al., 2016).

#### Resistance to verticillium wilt

Verticillium wilt is mainly caused by *Verticillium dahliae* or *Verticillium alboatrum*, two soil-borne vascular fungal pathogens that severely affect cotton production (Klosterman et al., 2009). After infection by *Verticillium dahliae*, the expression of *GhADF6*, a gene homologous to *AtADF6* found in upland cotton (*Gossypium hirsutum*), was downregulated in root epidermal cells. Silencing of *GhADF6* increased the abundance of microfilaments in root epidermal cells, and the plants showed enhanced resistance to *Verticillium dahlia*. Thus, *GhADF6* likely plays a negative regulatory role with respect to cotton verticillium wilt resistance (Sun et al., 2021). Taken together, these findings highlight the complex regulatory roles of *ADF* genes regarding plant defense against fungal diseases.

### Bacterial stress

#### Participation in Innate immunity caused by bacterial MAMP

Innate immunity is the first line of host defense against microbial invasion and is evolutionally conserved in all multicellular organisms, which is activated by pattern-recognition receptors (PRRs) that recognize microbe-associated molecular patterns (MAMPs) (Deng et al., 2020). MAMPs mediated microfilament rearrangement, as featured by increased abundance and remodeling of microfilament, plays an important role in plant innate immune signal transduction (Henty-Ridilla et al., 2014). Within minutes of treatment with a bacterial MAMP, elf26 (a conserved 26-amino acid peptide from bacterial elongation factor), a dose- and time-dependent increase in actin filament abundance was detected in epidermal cells throughout the *Arabidopsis* hypocotyl. However, actin architecture and dynamics in an *atadf4* mutant fail to respond to elf26 treatment, suggested that AtADF4 plays a key role in modulating actin dynamics by participating in innate immune signal transduction caused by bacteria in plants (Henty-Ridilla et al., 2014).

#### Resistance to Pst DC3000 expressing AvrPphB

*Pseudomonas syringae* pv *tomato* (*Pst*) is a hemibiotrophic bacterial pathogen. The *Arabidopsis* mutant *atadf4* shows abnormal microfilament dynamics and increased susceptibility to that of *Pst* DC3000 expressing the incompatible effector AvrPphB, but not to strains expressing AvrRps2 or AvrB. Moreover, a transgenic experiment showed that *AtADF4* is able to restore the resistance that is compromised in the *atadf4* mutant, thereby indicating that *AtADF4* is required for the resistance to *Pst* DC3000 expressing AvrPphB (Tian et al., 2009). *RPS5* (resistance to *Pseudomonas syringae* 5) is a gene that encodes a resistance protein capable of recognizing AvrPphB and activating downstream defense signals in *Arabidopsis* (Chisholm et al., 2006). Subsequent studies have reported that the increased susceptibility of *atadf4* mutant to *Pst* DC3000 expressing AvrPphB is associated with decreased *RPS5* expression, suggesting that *AtADF4* may regulate plant resistance to *Pst* DC3000 expressing AvrPphB via the coordinated regulation of microfilament dynamics and *R*-gene transcription (Porter et al., 2012).

#### Participation in interaction with rhizobia

Rhizobia is a class of gram-negative soil bacteria that includes *Rhizobium*, *Bradyrhizobium*, *Sinorhizobium*, and *Azorhizobium.* These bacteria can form symbiotic nitrogen-fixing nodules with leguminous plants and increase nitrogen fixation in arable fields by as much as 30% (Mus et al., 2016). *PvADFE* is one of the nine *ADF* genes found in common bean, primarily expressed in roots and nodules inoculated with *Rhizobium tropici* (Ortega-Ortega et al., 2020). In addition, *PvADFE* silencing increases the number and size of nodules and enhances nitrogen fixation activity. Conversely, the overexpression of this gene resulted in the opposite phenotype. In addition, the expression levels of two genes related to nodulation development and signaling, *NIN* and *ENOD2*, were significantly decreased in the roots of plants overexpressing *PvADFE*, thereby indicating that *PvADFE* plays a negative regulatory role in rhizobial infection and nodulation of common bean (Ortega-Ortega et al., 2020).

### Viral stress

Viruses are molecular parasites that complete the entire life cycle by utilizing the resources of host cells. Many crucial functions of plants are affected by viruses, including nutrient absorption, nutrient translocation, photosynthesis, growth, and development (Gergerich and Dolja, 2006). In an infected plants, virus-encoded movement proteins and cellular factors allow viruses to move within infected cells (local movement) and long distances through the vascular system (systemic movement) (Garcia-Ruiz, 2018). A great deal of attention is given to understanding the fundamental mechanism of viral infections as well as factors involved in gene regulation during viral infections (Garcia-Ruiz, 2018). Microfilament has been reported to play an important role in the process of virus infection (Chen et al., 2010; Tilsner et al., 2012; Porter and Day, 2016).

Soybean mosaic virus (SMV), which belongs to the Potyvirus genus, is one of the most prevalent and destructive viral pathogens in soybean cultivation regions around the world. Mosaic and necrosis symptoms are common on the leaves of soybean plants that are infected with SMV (Hill et al., 2007). The P3 protein of SMV (SMV-P3) plays a major role in its replication and movement, and also responsible for symptom development in SMV-infected plants (Hajimorad et al., 2018). SMV-P3 exhibits strong variability and complex functionality, which is consistent with the symptoms of soybean mosaic disease (Hajimorad et al., 2018). By screening soybean cDNA library, Lu et al. (2015) found that an ADF, GmADF2, interacts with SMV-P3, and this interaction is further confirmed using bimolecular fluorescence complementation assay. Further experiments showed that the interaction between GmADF2 and SMV-P3 is occurred in both the cytomembrane and cytoskeleton of plant cells, indicated the GmADF2 was trailed by SMV-P3 (Lu et al., 2015). These results suggested that GmADF2 is an important host factor for SMV-P3 and may promote its intercellular movement, thus plays a crucial role for the virus to establish infection (Lu et al., 2015).

## Role of *ADF* genes in abiotic stress resistance

**Figure 1:**
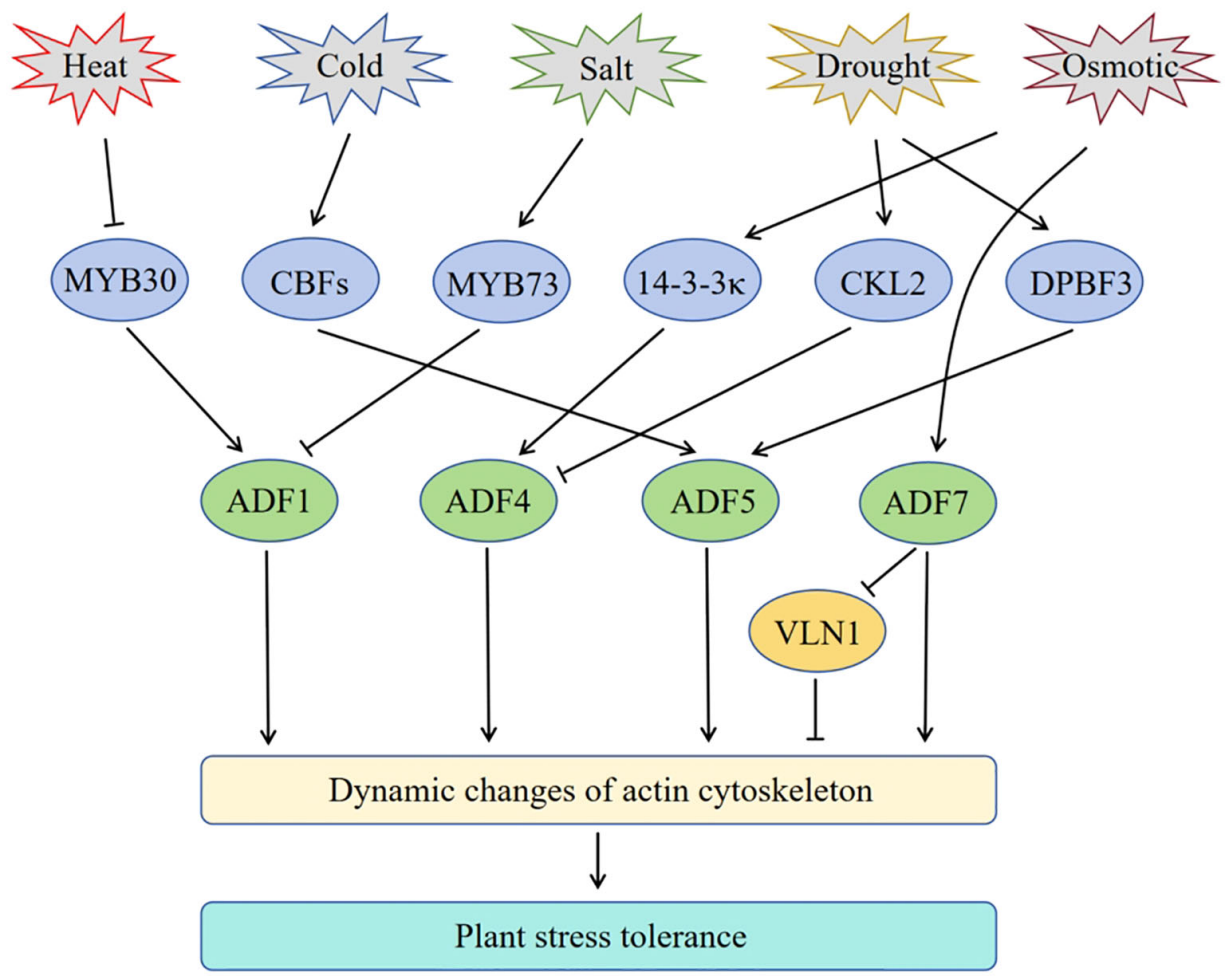
Schematic of the involvement of Arabidopsis *ADF* genes in plant response to abiotic stress. Arrows represent positive regulation, and bar ends mean inhibitory action.

In addition to biotic stresses, abiotic stresses like cold, heat, drought, salinity, flooding and nutrient deficiency are the major limiting factors for crop yields (Saijo and Loo, 2020). Abiotic stress factors can individually or collectively affect plant growth and development (Zhu, 2016). Plant *ADF* genes are widely involved in various abiotic stress responses (**Figure 1**). However, their modes of response and functions vary among plant species and tissues (**Table 2**). In this section, we will review the functions and molecular regulatory mechanisms of plant *ADF* genes in regulating abiotic stress in plants.

**Table 2:** *ADF* genes involved in abiotic stress whose functions have been elucidated.

Gene	Organism	Inducing factor	Function	Upstream regulator or downstream target	References
*AtADF1*	*Arabidopsis*	Heat	Negative regulator of heat tolerance	Regulated by AtMYB30	Wang L. et al., 2023
*BrADF1*	Chinese cabbage	Heat	Negative regulator of heat tolerance	NG	Wang B. et al., 2023
*AtADF5*	*Arabidopsis*	Cold	Positive regulator of cold tolerance	Regulated by CBF	Zhang et al., 2021
*TaADF16*	Wheat	Cold	Positive regulator of cold tolerance	Induces expression of cold-related genes	Xu et al., 2021
*DaADF3*	Antarctic hairgrass	Cold	Positive regulator of cold tolerance	NG	Byun et al., 2021
*AtADF1*	*Arabidopsis*	Salt	Positive regulator of salt tolerance	Regulated by AtMYB73	Wang et al., 2021
*SaADF2*	Smooth cordgrass	Salt and drought	Positive regulator of salt and drought stress tolerance	NG	Sengupta et al., 2019
*AtADF4*	*Arabidopsis*	Osmotic stress	Negative regulator of osmotic tolerance	Regulated by 14-3-3κ	Yao et al., 2022
*AtADF7*	*Arabidopsis*	Osmotic stress	Positive regulator of osmotic tolerance	Inhibits expression of VLN1	Bi et al., 2022
*AtADF4*	*Arabidopsis*	Drought	Positive regulator of drought tolerance	Regulated by CKL2	Zhao et al., 2016
*AtADF5*	*Arabidopsis*	Drought	Positive regulator of drought tolerance	Regulated by DPBF3	Qian et al., 2019
*PeADF5*	*Populus euphratica*	Drought	Positive regulator of drought tolerance	Regulated by PeABF3	Yang et al., 2020
*OsADF3*	Rice	Drought	Positive regulator of drought tolerance	NG	Huang et al., 2012

### Temperature stress

#### Cold stress

Cold stress, including chilling (cold temperatures of above 0°C) and freezing stress (below 0°C), causes plant growth to slow down, stagnate, and retrogress, thereby reducing the yield (Zhang et al., 2019). Aside from membrane rigidification, ROS accumulation, protein destabilization, and metabolic disequilibrium, cold stress has also been reported to disrupt the microfilament of plant cells and interfere with all cellular processes (Fan et al., 2015; Liu et al., 2018). The expression profiles of *AtADF* genes in response to temperature stress had been reported to diversely among different groups. The expression levels of *AtADF* genes of Group I, III, and IV have been found to be significantly induced by cold or heat stress, with two group III *ADF* genes (i.e., *AtADF5* and *AtADF9*) responding the most strongly (Fan et al., 2015; Fan et al., 2016). In *Arabidopsis* plants exposed to cold stress, the survival rate of *atadf5* mutants significantly decreased relative to the wild type. Moreover, mutant plants showed disordered actin cytoskeleton in root epidermal cells, suggesting that *AtADF5* plays an important role in mediating cold stress tolerance in *Arabidopsis* (Zhang et al., 2021). In the face of cold stress, plants depend on C-repeat binding factors (CBFs) as their key molecular switches (Liu et al., 2018). CBFs can activate the expression of *AtADF5* by binding to CRT/DRE elements in its promoter, and AtADF5 can in turn regulate dynamic changes in the actin cytoskeleton to modulate the cold response of *Arabidopsis* plants (Zhang et al., 2021). In freezing-tolerant wheat cultivars, the *ADF* gene *Wcor719* can be specifically induced by exposure to low temperatures, while the expression levels of this gene do not significantly change in freezing-sensitive cultivars. Moreover, its expression level is also insensitive to high temperature, salt stress, mechanical damage, and abscisic acid (ABA) (Danyluk et al., 1996; Ouellet et al., 2001). In contrast, wheat *TaADF4* is induced by heat stress, but its expression levels are significantly decreased in response to low temperature or salt stress (Zhang et al., 2017). A genome-wide analysis showed that 25 *TaADF* genes exist in the genome of the “Chinese Spring” wheat cultivar, and that cold stress can affect the expression levels of seven *TaADF* genes, six of which (i.e., *TaADFs 13*, *16*, *17*, *18*, *21*, and *22*) are upregulated (Xu et al., 2021). In *Arabidopsis*, the heterologous expression of *TaADF16*, the most highly expressed and upregulated *ADF* gene in response to cold stress, can enhance plant cold stress resistance by accelerating ROS scavenging and by altering osmotic regulation in cells (Xu et al., 2021). Moreover, the expression levels of seven cold stress-responsive genes were found to be significantly higher in a *TaADF16*-overexpressing line than in the wild type regardless of whether the transgenic *Arabidopsis* plants were exposed to cold conditions. This indicates that the overexpression of *TaADF16* can generally induce the expression of cold-related genes (Xu et al., 2021). Antarctic hairgrass is the only monocotyledonous flowering plant in Antarctica and its genome contains eight *ADF* genes. Cold stress can induce the expression of five *DaADF* genes, with *DaADF3* showing the most significant cold stress response (Byun et al., 2021). In rice, plants that overexpress *DaADF3* exhibit improved cold stress resistance, as measured via survival rate, leaf chlorophyll content, and electrolyte leakage along with changes in microfilament organization in the root tips (Byun et al., 2021).

#### Heat stress

Heat stress is commonly defined as the increase in temperature beyond a specific threshold level for a duration that is adequate to induce irreversible harm to the growth and development of plants (Bita and Gerats, 2013). The effects of heat stress on plants and cells are numerous. For example, high temperatures alter membrane fluidity and denaturate proteins which impair enzyme function (Malerba et al., 2010). Recently, *ADF* genes have also been found to be involved in plant tolerance to high temperatures. For example, *AtADF1* expression was repressed by high temperatures, and *atadf1* mutant seedlings exhibited greater actin filament stability and faster growth than the wild type (Wang L. et al., 2023). Conversely, *AtADF1* overexpression showed the opposite phenotype. Further experiments revealed that *AtADF1* transcription is regulated by AtMYB30, a key transcription factor involved in responses to various forms of abiotic stress, including heat (Liao et al., 2017). This finding indicates that *AtADF1* is a target gene in the AtMYB30-mediated plant response to abiotic stress (Wang B. et al., 2023). Similarly, the authors found that *BrADF1* from Chinese cabbage (*Brassica rapa*), a gene that is highly homologous to *AtADF1*, regulates F-actin dynamics and plant tolerance to heat stress in a manner similar to that of *AtADF1* (Wang L. et al., 2023).

#### Salt stress

Salt stress refers to the adverse effect of excessive soluble salts in soil on plant growth and development, which has both osmotic and ionic or ion-toxicity effects on cells (Zhu, 2016). More than one third of the world’s irrigated lands are affected by salinization, a worldwide problem that threatens the growth and yield of crops (Zhao et al., 2020). In *Arabidopsis*, the expression levels of *AtADF1* rapidly increase in response to salt stress, and the survival rate of the *atadf1* mutant decreases significantly compared with the wild type under salt stress. Moreover, mutant plants exhibit cytoskeletal changes, including increased microfilament bundles in cells, while *AtADF1-*overexpressing plants exhibit opposite macroscopic and microscopic phenotypes. These results indicate that *AtADF1* positively regulates plant salt tolerance by promoting actin depolymerization (Wang et al., 2021). Moreover, AtMYB73 is a negative regulatory factor for salt stress in *Arabidopsis* (Kim et al., 2013), and further experiments have revealed that AtMYB73 negatively regulates *AtADF1* expression (Wang et al., 2021). Therefore, *AtADF1* is likely an important player in the AtMYB73-mediated salt stress response pathway (Wang et al., 2021). Smooth cordgrass (*Spartina alterniflora*) is a perennial grass halophyte that has adapted to salt and drought conditions owing to specific alleles for genes involved in stress tolerance (Baisakh et al., 2008). The *SaADF2* of smooth cordgrass is homologous to the *OsADF2* of rice. Although the sequence similarity between their proteins exceeds 95%, six amino acid differences (i.e. the 6^th^ Ser, 19^th^ Asp, 25^th^ Leu, 118^th^ Gln, 132^nd^ Pro and 133^rd^ Thr in OsADF2 were substituted by Thr, Asn, His, His, Ser and Ser in SaADF2, respectively) may responsible for the substantial differences in their three-dimensional structures (Sengupta et al., 2019). Biochemical analysis revealed that SaADF2 displays greater actin-binding affinity and can depolymerize microfilaments more efficiently than OsADF2, which enhances cellular actin dynamics in cells. In rice, *SaADF2* overexpression engenders greater drought and salt tolerance compared with that in the wild type and *OsADF2*-overexpression lines (Sengupta et al., 2019). A detailed biochemical investigation is required to determine the specific amino acid(s) that play a critical role in ADF’s biochemical activity. This knowledge could present an opportunity to utilize genome-editing technology for performing site-specific mutations, enabling the manipulation of ADF activity in crop breeding practices for enhancing stress tolerance.

#### Osmotic stress

Osmotic stress, often caused by drought and high salinity, occurs when soil contains excess soluble salt that prevents water absorption of plants (Yoshida et al., 2014). Microfilament cytoskeleton had been confirmed to participates, and plays a crucial role, in responses to osmotic stress in plants (Wang et al., 2010). In *Arabidopsis* seedlings subjected to osmotic stress, expression level of *AtADF4* considerably increased. In addition, the survival rate of *atadf4* mutants was higher than that of the wild type, while the survival rate of *AtADF4*-overexpressing lines decreased. Thus, these results indicate that *AtADF4* plays a negative regulatory role in the plant response to osmotic stress (Yao et al., 2022). Further experiments demonstrated that a phosphopeptide-binding protein, 14-3-3κ, acts as an upstream regulator of *AtADF4* to regulate the *Arabidopsis* response to osmotic stress (Yao et al., 2022). Root hairs are important organs for plants to absorb nutrients and water. Osmotic stress can induce *AtADF7* expression, which in turn is responsible for inhibiting the expression of the actin-bundling protein VILLIN1 (VLN1) in root cells, thereby reducing microfilament bundles in root cells and promoting root hair growth. These findings indicate that the AtADF7–VLN1 pathway is essential for root hair formation under osmotic stress tolerance, and plays critical role in enhancing plant osmotic stress tolerance (Bi et al., 2022).

#### Drought stress

Drought is an adverse environmental stress that hampers normal growth, disrupts water relations, and decreases water-use efficiency in plants. To adapt to drought stress, plants have developed intricate mechanisms, one of which involves regulating the opening and closing of stomata (Saradadevi et al., 2017). Stomata are pores found on the epidermis of aerial parts of plants and are responsible for absorbing carbon dioxide and releasing water vapor (Jiang et al., 2012). The stomatal aperture is finely tuned to prevailing environmental conditions by a pair of guard cells surrounding each pore (Schroeder et al., 2001). Stomatal movement mediated by ABA is particularly important for plant adaptations to drought conditions. Microfilament dynamics play a crucial role in regulating the opening and closing of the stomata, involving the alteration of the radial orientation of the actin filaments during open stomata changes to a longitudinal orientation characteristic of closed stomata during stomatal closure (Zhao et al., 2011).

In *Arabidopsis*, *AtADF1* overexpression leads to disorganized microfilament bundles in guard cells, in turn resulting in abnormal stomatal closure following ABA treatment (Dong et al., 2001). *Arabidopsis* casein kinase 1-like protein 2 (CKL2) plays an important regulatory role in ABA- and drought-induced stomatal closure. CKL2 can inhibit the depolymerization activity of AtADF4 via phosphorylation, thereby rendering the microfilament cytoskeleton more stable in guard cells and regulating stomatal opening or closing (Zhao et al., 2016). ABA and drought stress also induce *AtADF5* expression in *Arabidopsis* seedlings. Compared with the wild type, an *atadf5* mutant showed reduced microfilament bundles in cells, delayed stomatal closure, intensified leaf dehydration, and decreased survival rates under drought conditions (Qian et al., 2019). Further biochemical experiments demonstrated that DPBF3, an ABA-responsive element-binding factor (ABF/AREB), can activate *AtADF5* expression via ABA-responsive core elements in its promoter region. AtADF5 regulates stomatal movement by modulating the rearrangement of microfilament structures through its F-actin bundling activity, which improves plants’ adaptability to drought stress. Therefore, AtADF5 is an important player in the ABF/AREB-mediated pathway facilitating plant responses to drought stress (Qian et al., 2019). In *Populus euphratica*, PeABF3 is a transcription factor involved in ABA signaling response and its expression is induced by drought and ABA. PeABF3 can activate the expression of *PeADF5*, which facilitates ABA-induced stomatal movement by promoting actin cytoskeletal rearrangement and enhancing drought resistance (Yang et al., 2020). Furthermore, in rice the exogenous application of ABA or various stress conditions induces *OsADF3* expression in the root tips and lateral roots. Moreover, the heterologous expression of *OsADF3* in *Arabidopsis* enhanced its drought tolerance, as evidenced by improved germination rate, primary root length, and survival rate. In addition, several drought-tolerance responsive genes are upregulated under drought stress, suggesting that *OsADF3* may exert regulatory effects upstream of these genes (Huang et al., 2012). The above studies indicate that plant *ADF* genes are important factors in regulating stomatal movement and play an important role in enhancing plant drought resistance. Further exploration and research on these drought resistant *ADF* genes and homologous *ADF* genes in other plants, especially crops, will provide important genetic resources for the cultivation of drought resistant crops.

## Summary and future prospects

As an important type of actin-binding protein, ADFs are widely involved in dynamic changes to the microfilaments of cells. Accordingly, they play a crucial role in plant growth, development, and stress response. In this study, we provide a systematic summary of the involvement of ADFs in the regulation of both biotic and abiotic stresses in plants. This includes the expression patterns of *ADF* genes in response to various stresses, their regulatory role with respect to plant stress responses, and the molecular mechanisms by which ADFs regulate stress tolerance. Research suggests that stress conditions not only directly regulate the transcription levels of *ADF* genes, but many transcription factors (including members of the MYB, ABF, and CBF TF families) are also involved in regulating the expression of *ADF* genes in response to different forms of stress. Furthermore, ADFs not only directly regulate the polymerization, depolymerization, and arrangement of microfilaments in cells, but also indirectly affect plant stress responses by influencing the expression of various other stress-related genes. Taken together, these results indicate that *ADF* participates in precise and complex mechanisms to regulate plant stress responses.

However, to date our understanding of the role of *ADF* genes in the regulation of plant stress responses remains insufficient. First, there is currently limited systematic information regarding the *ADF* gene family in plants, since data exists for fewer than 20 plant species. Previous studies have shown that *ADF*s exist in land plants as members of diversified gene families, and their expression patterns and biochemical activities exhibit obvious inter-group specificity. Genomic and transcriptomic studies provide a convenient way to comprehensively identify and characterize the *ADF* gene family in additional plant species. Moreover, systematic analysis of the plant *ADF* gene family will provide important information for further investigation of the biological functions of different *ADF* genes. Second, previous studies have found that the expression levels of many plant *ADF* genes change in response to stress, which suggests that *ADF* genes play an important role in plant stress responses. At the molecular regulation level of the *ADF* gene regulation of stress responses, *Arabidopsis thaliana* has received an overwhelming share of the research attention and has made significant progress in understanding *ADF* genes in this model system. However, there has been limited research on other plants, especially on agriculturally important crops, in which the specific functions and molecular regulatory mechanisms of *ADF* genes remain largely unclear. This hinders their application for crop improvement. Future in-depth study of stress-related *ADF* genes in crop species is critical for molecular breeding and genetic engineering.

Finally, studies of *SaADF2* in salt- and drought-tolerant smooth cordgrass suggest that some key amino acids in ADF influence its biochemical activity, and can thereby be manipulated to exert stronger regulatory effects on specific plant stress responses. This study suggests that there may be beneficial *ADF* alleles in plant species with strong stress resistances that may enhance crop resilience. In the future, detailed investigation of these genes and exploration of advantageous protein variant sites may make it possible to use genome-editing techniques to modify *ADF* genes for stress-tolerant crop breeding.

## Author contributions

YS: Funding acquisition, Writing – original draft, Writing – review and editing. MS: Writing – original draft. DW: Writing – original draft. YG: Writing – original draft. QS: Writing – original draft. PL: Writing – original draft. JY: Investigation, Writing – original draft. PC: Investigation, Writing – original draft. SG: Writing – review and editing.
